# Geological Affinities of Thermal Springs

**Published:** 1859-01

**Authors:** 


### Editors' Table.

Geological Affinities of Thermal Springs.—In a previous article on the subject, (July, 1858,) we alluded to the affinity of volcanic action to the thermalism of springs, and stated that the sublimates, or substances of volcanos when converted into a gaseous and fluid state, give us nearly all the ingredients of mineral and thermal springs. Beginning with the proposition advanced by Stefft, that, "generally mineral springs follow the course of mountains composed of igneous rocks," and the modification advanced by Bischoff, that "we shall find it impossible to avoid recognizing a relation between elevations of Plutonic masses, the upraising of Neptunian formations, and thermal springs," we come to the more positive and distinctly expressed belief of Keferstein and Van Hoff, that hot springs constantly accompany volcanic regions. Dr. Daubeny, in his treatise on Active and Extinct Volcanos, has placed this subject in a very clear point of view, and with an array of facts and well-conducted argument which can hardly fail to secure the opinion of the reader on his side.

Wide as is the geographical range of volcanos, that of thermal waters, as we shall soon see, is not less so. In the New, as in the Old World, they are found in close association, one with the other, and evince, in many instances, analogous modes of exhibition, and, as said above, almost identity of products. The eruptions of some volcanos, as those of Peru and Quito, are destructive, not by the lava, but by the water and enormous streams of mud thrown out from them. The water of one of the boiling mud cauldrons of Iceland, analyzed by Professor Bunsen, contained the same substances of which the lava from Mount Vesuvius was composed, with the exception of protoxide of iron in the latter, which was absent from the former.

Travelers who visit Iceland, when they see Hecla with its volcano, and the neighboring hot springs of the Geysers and Strokr throwing up lofty columns of water and steam, and when they hear subterranean noises like thunder, while the ground vibrates and trembles under their feet, as if it were about to open and engulf them in the abyss beneath, are forcibly impressed with the resemblances between volcanic and thermal phenomena. These are made the more striking by the explosive force and "the frightful violence" with which the water is ejected, and by the intermittent character of the eruption. Large stones thrown into the spring were projected to such a height that they could hardly be seen by the naked eye. Many were so perfectly vertical in their ascent that they fell back again into the pipe of the spring; and they seemed, in their subsequent projections, to answer the purpose of balls for this gigantic spring to play with.

The Hot Springs of Furnas, in the island of St. Michael, one of the Azores, exhibit many phenomena similar to those of Iceland, and, at the same time, their connection with volcanos.

The water in the larger spring, or Caldeira, boils with violence, and emits, at short intervals, loud explosions, which are succeeded by a distinct rise of the water in its containing basin. During this latter period a loud hissing noise is heard, with an escape of great quantities of sulphuretted hydrogen gas, steam, and sulphurous acid. All of these are volcanic products likewise. Silica, so predominant an ingredient in the hot springs of Iceland, is also found in those of St. Michael. Sulphur abounds near the springs. Contiguous to the principal hot spring, or Caldeira, there is a hill fifty feet in height, the side of which having fallen in, opens to view a deep cavern, frightful by its extent, its steam, and gaseous exhalations, and the tremendous noise issuing from it.

Similar associations between volcanic action and thermal springs are found on the slope of Vesuvius, and around the Bay of Naples, (the Phlogræan Fields,) in the region of Mount Etna, and among the volcanos of the New World. A still more striking example was furnished by the volcano of Jurillo, in Mexico, which was suddenly protruded from the great plain of Malpais to the height of 1600 feet, in a single night, in the year 1759. The space, now the site of a volcano, was occupied by fertile fields of sugar-cane and indigo, and watered by two streams, Cuitimba and San Pedro, which disappeared during the eruption; losing themselves below the eastern extremity of the plain, and reappearing as hot springs at its eastern limit. The appearance of the volcano of Jurillo was preceded, during two months, by earthquakes, accompanied by the issue of flames from the ground and the ejection of burning rocks to a prodigious height.

In further confirmation of the view now taken, we may state that the constituents of lava, pumice and other volcanic products, are often the same as those deposited by thermal springs; and that igneous rocks are decomposed and their chief constituents held in solution by these springs. As remarked by De la Beche, many thermal, and at the same time mineral springs, appear to be only the condensation of vapors and gases effected in portions of fissures from volcanic vents, after the temperature has become sufficiently lowered. Rocks formed by deposits from thermal springs have, as pointed out by Menge, many of the characters of basalt, porphyry, wacke, tufa, and obsidian—recognized volcanic products. To the same thermal origin, Jameson and others attribute precious opal, common opal, and jaspers of various descriptions.

To the emanations, in the form of vapors, and to mineral waters, Elie de Beaumont refers the origin of mineral veins, the various facts relating to which we are enabled, by this hypothesis, to comprehend, especially the development of those chemical affinities which have long been observed to influence the manner in which the metals are associated. Substances which are usually brought together have much in common between them; and they often exhibit quite analogous properties. Such are nickel and cobalt; iron and manganese; antimony and arsenic; silver and lead. With respect to the vapors and gaseous substances thus discharged from volcanos, and found in mineral waters, the writer just named has pointed out nineteen substances contained alike in them and in mineral veins, viz., *potassium*, *sodium*, *calcium*, *aluminum*, *manganese*, *iron*, *cobalt*, *lead*, *copper*,

*hydrogen*, *silicon*, *carbon*, *borax*, *arsenic*, *nitrogen*, *selenium*, *sulphur*, *oxygen*, and *chlorine*. The substances found in mineral waters and veins, and not hitherto noticed in volcanic emanations, are, *lithium*, *borium*, *strontian*, *magnesium*, *phosphorus*, *iodine*, *bromine*, and *fluorine*.

As exhibiting both the origin of common and thermal springs and their chemical and geological relations, the paper by Bunsen, "On the Intimate Connection Existing Between the Pseudo-volcanic Phenomena of Iceland," in one of the Cavendish Society volumes, may be consulted with advantage. He tells us, that the active volcanos, "upheaval on fissures, intersect the whole island in a parallel system of longitudinal lines, whose north-eastern extremity corresponds perfectly with the parallel expansion of the principal valleys and elevated ridges of the main system, and with the numerous volcanic fissures and dykes with which the country abounds, while the fumaroles and thermal systems appear to be likewise connected with the main direction." This last may be traced in lines passing transversely over mountains and valleys. "This circumstance, and the common occurrence of solfataras, suffiones, thermal springs and geysers, and, still more, the intimate connection existing between the phenomena of decomposition, to which they give rise, lead, even on a superficial observation, to the conclusion that all these phenomena are merely to be regarded as one and the same fundamental cause."

While the evidence is thus cumulative in favor of the intimate relations, as effects of a common cause, between volcanos and thermal springs, we cannot, on the other hand, overlook the decidedly adverse opinion of Humboldt, who asserts that hot springs burst out of the most diversified mineral strata. Among the hottest of all the perennial springs which have yet been observed, are those discovered by this celebrated traveler and savant, and which flow remote from all volcanos. He refers to the *Aguas Calientes de las Trincheras*, between Porto Cabello and New Valencia, and to the *Aguas de Cimangillas Guanaxuesta*, in Mexico. The first-mentioned spring issues from granite, and its temperature is 162° 5′ F.; the second from basalt, showed 173° 5′ F. But this broad assertion is not so much at variance with the view which we have presented to our readers, especially when we receive the amplification given to it by Daubeny, viz.: that hot springs, in a very large proportion, arise from rocks, which, in their general aspect and structure, attest the operation of volcanic forces at one time or another. Even if we should still admit that there is a large proportion of thermal springs which appear to be quite remote from volcanos, either active or extinct, we shall find, nevertheless, that great numbers of these springs rise in primitive mountain chains, which, although they may not present on their surface volcanic products, bear undoubted marks of igneous origin. The thermal waters of the Alps do not, writes Balkewell, rise in the upper strata, but spring out of the lower primary rocks. The range from the Simplon, through Savoy, to the confines of France, and even to Dauphiny, is in nearly the same direction. Earthquakes occur in the Upper Valais, where the temperature of the water is highest. The mountains in the above range are placed at or near to one common source of heat, to the agency of which they owe their original elevation. All along the southern declivity of the Alps, as pointed out by Daubeny, a continued chain of hot springs emerges from the line of junction of the primitive with the newer rocks, which can be traced from the foot of Mount St. Blanc and the Great St. Bernard to the volcanic products of the Euganean Hills, in which are found the thermal waters of St. Didier, Acqui, Pelegrino, Bormio, Masimo, Caldero, Abano, Balttaglia, and Monte Orlone.

Professor Forbes points out, as remarkable, the connection of the appearance of hot springs with granite in the region of the Pyrenees. Their abundance increases as we advance eastward in the range. At Ax, as we are told by the same writer, this most remarkable thermal site, reported to be the most prolific in Europe, confirms "the general principle, *that hot springs take their origin* at the boundary *of granite.*" Ax is situated on the River Ariége, several leagues above Ussac. We pass from limestone to slate, and from slate to granite, precisely at Ax. In this town alone, seventy or eighty hot springs take their origin; scarcely an opening in the ground can be dug to any depth without water making its appearance. The fountains in the market-place are supplied with it; manufactories are worked by it; even culinary operations are performed by it; and all this, continues Mr. Forbes, in addition to the supply of innumerable baths, with such varieties of the water as are most highly mineralized. The chief groups of springs are at the market-place, and of these the most remarkable is the *Source des Canons*, the water of which has a temperature of 168° F., and is nearly pure.

The springs of Thorez, in the Pyrenean group, are among the hottest in Europe, being from 166° to 171° 5′ F. Arago declared the spring at Chandes Aigues, in Auvergne, to be the hottest in Europe of those unconnected with recent volcanic action. He quotes it at 176° F., or 80° Cent. Among the springs of the Pyrenees those of Bagnères-de-Luchon issue from the granite within a few feet of each other, although, curiously enough, they differ in their temperature as well as in other respects.

Captain Newbold asserts that a majority of the springs of India, which can be strictly called thermal, occur on or near lines of great faults, occasioned by the upheaval of Plutonic rocks. A similar remark may be extended to the most celebrated of the hot springs of Asia Minor. So, likewise, in many of the European islands, the connection between volcanos and igneous upheavals on the one side, and thermal springs on the other, is of frequent occurrence. Examples of the contiguity of some springs to remarkable dislocations of strata are presented at Aix, and Carlsbad, and at Pfeffers, and other thermal springs in Switzerland, and at Clifton and Malvern in England.

Coincident with the preceding views are those entertained by Dr. Wm. B. Rogers, in a paper on "The Connection of Thermal Springs with Anticlinal Axes and Faults." Of *fifty-six* thermal springs, chiefly in the great limestone valley of Virginia, *forty-six* of this number are situated on or adjacent to anticlinal axes; *seven* on or near lines of faults and inversion; and *three*, the only group of this kind yet known in Virginia, close to the point of junction of the Apalachian with the Hypogene (primitive) rocks.

In conclusion, we shall point to the fact of the connection between earthquakes and thermal springs, which we have shown to exist between the latter and volcanos All these are referable to a common cause, great heat in the interior of the earth, and their differences are explicable by the greater or less facility of its passage to the surface. Thermal and mineral springs are abundant in countries of earthquakes, as well as of active volcanos. We might cite various instances of the effects of earthquakes on thermal springs, in sometimes stopping for a while their flow, sometimes rendering them turbid, and, again, changing their temperature.

On a future occasion we shall ask our readers to accompany us in our tour in quest of thermal springs. The search will be rich in its results.

